# VFM-SSL-BMADCC-Framework: vision foundation model and self-supervised learning based automated framework for differential cell counts on whole-slide bone marrow aspirate smears

**DOI:** 10.3389/fmed.2025.1624683

**Published:** 2025-09-24

**Authors:** Shirong Zhou, Longrong Ran, Yuanyou Yao, Xing Wu, Yao Liu, Chengliang Wang, Zhongshi He, Zailin Yang

**Affiliations:** ^1^College of Computer Science, Chongqing University, Chongqing, China; ^2^Chongqing Key Laboratory of Translational Research for Cancer Metastasis and Individualized Treatment, Department of Hematology-Oncology, Chongqing University, Chongqing, China; ^3^Center for Hematology, Southwest Hospital, Army Medical University, Chongqing, China

**Keywords:** whole-slide bone marrow aspirate smears, differential cell counts, vision foundation model, self-supervised learning, texture

## Abstract

**Background:**

Differential cell counts (DCCs) on bone marrow aspirate (BMA) smear is a critical step in the diagnosis and treatment of blood and bone marrow diseases. However, manual counts relies on the experience of pathologists and is very time-consuming. In recent years, deep learning-based intelligent cell detection models have achieved high detection accuracy on datasets of specific diseases and medical centers, but these models depend on a large amount of annotated data and have poor generalization. When the detection task changes or model is applied in different medical centers, we need to re-annotate a large amount of data and retrain the model to ensure detection accuracy.

**Methods:**

To address the above issues, we designed an automated framework for whole-slide bone marrow aspirate smear differential cell counts (BMADCC), called VFM-SSL-BMADCC-Framework. This framework only requires whole-slide images (WSIs) as input to generate DCCs. The vision foundation model SAM, known for its strong generalization ability, precisely segments cells within the countable regions of the BMA. The MAE, pre-trained on a large unlabeled cell dataset, excels in generalized feature extraction, enabling accurate classification of cells for counting. Additionally, TextureUnet and TCNet, with their powerful texture feature extraction capabilities, effectively segment the body-tail junction areas from WSIs and classify suitable tiles for DCCs. The framework was trained and validated on 40 WSIs from Chongqing Cancer Hospital. To assess its generalization ability across different medical centers and diseases, correlation tests were conducted using 13 WSIs from Chongqing Cancer Hospital and 5 WSIs from Southwest Hospital.

**Results:**

The framework demonstrated high accuracy across all stages: The IoU for region of interest (ROI) segmentation was 46.19%, and the accuracy for tile of interest (TOI) classification was 90.45%, the Recall75 for cell segmentation was 99.01%, and the accuracy for cell classification was 77.92%. Experimental results indicated that the automated framework had excellent cell classification and counts performance, suitable for BMADCC across different medical centers and diseases. The differential cell counts results from all centers were highly consistent with manual analysis.

**Conclusion:**

The proposed VFM-SSL-BMADCC-Framework effectively automates differential cell counts on bone marrow aspirate smears, reducing reliance on extensive annotations and improving generalization across medical centers.

## 1 Introduction

Bone marrow aspirate smear differential cell counts (BMADCC) plays a crucial role in diagnosing and treating hematologic malignancies. Specifically, it involves the process of pathologists determining the proportions of various cell types within the bone marrow.

BMADCC helps diagnose acute lymphoblastic leukemia (ALL) (1), acute myeloid leukemia (AML) (2), angioimmunoblastic T-cell lymphoma (AITL) (3), Burkitt lymphoma (Burkitt) (4), chronic lymphocytic leukemia (CLL) (5), chronic myelogenous leukemia (CML) (6), chronic myelomonocytic leukemia (CMML) (7), classic Hodgkin lymphoma (cHL) (8), diffuse large B-cell lymphoma (DLBCL) (9), essential thrombocythemia (ET) (10), follicular lymphoma (FL) (11), mantle cell lymphoma (MCL) (12), mucosa-associated lymphoid tissue lymphoma (MALT) (13), multiple myeloma (MM) (14), NK/T-cell lymphoma (NKTL) (15), prolymphocytic leukemia (PLL) (16), and immune thrombocytopenic purpura (ITP) (17), and other hematologic malignancies.

Traditional BMADCC is performed manually by pathologists using a microscope. Considered as a gold standard, this method is widely applicable for diagnosing and monitoring various blood and bone marrow diseases (18). However, it has two main drawbacks: (1) Labor-intensive: Manual counts is a time-consuming and labor-intensive task, requiring pathologists to spend significant amounts of time. Long-time continuous working may affect the accuracy of cell classification and counts. (2) Subjectivity: The inconsistency in DCCs experience levels among different pathologists leads to subjective bias (19).

With the rapid development of deep learning, vision models based on deep learning have shown excellent performance in image processing tasks such as object detection, semantic segmentation, and image classification. These models have enabled quantitative routine tasks in computer-aided diagnosis, thereby accelerating the process, reducing bias, and improving the consistency of results (20–22). To address the issues associated with manual counts, many researchers (23–29) have applied deep learning algorithms to achieve automated BMADCC. This has significantly improved the accuracy and efficiency of DCCs, holding great theoretical and practical value. These studies can be categorized into two main methods according to their processes: Tile-based counts methods and WSI-based counts methods.

Tile-based counts methods require manually slicing the WSIs into tiles (i.e., square bone marrow images), selecting the appropriate tiles as input for DCCs, and then performing cell detection and classification. Wang et al. (23) utilized the Faster-RCNN object detection algorithm and feature pyramid network to detect 6 types of cells within tiles. However, the cell detection model’s classification accuracy still needed improvement. In contrast, Chandradevan achieved higher overall accuracy by using a separate cell classification model (24). They developed a two-stage system for standard clinical cell classification, manually selecting tiles with a large number of cells from BMA, detecting all cells as single category objects using Faster-RCNN, and then classifying the cells with VGG. Yu et al. (25) applied deep convolutional neural networks to automatically detect and classify bone marrow nucleated cells within tiles. These methods require manual selection of optimal tiles, making the process slow and unsuitable for full-process DCCs, which limits their scalability to clinical diagnostic work.

Compared to Tile-based counts methods, WSI-based counts methods do not require manual selection of tiles. Instead, they automatically identify tiles of interest (TOI) from WSIs, detect cells within these tiles, and finally classify and count these cells, achieving intelligent full-process DCCs. These methods can be further categorized based on the approach of tile extraction: Grid-based counts methods and ROI-based counts methods.

Grid-based counts methods directly slices WSIs into uniformly sized tiles, then selects TOIs from these tiles. Tayebi et al. (26) developed an end-to-end automated bone marrow cytology system that slices WSIs into uniform tiles, uses DenseNet121 for binary classification to distinguish between TOI and Non-TOI, and then employs YOLOv4 for cell detection and classification within the TOIs. This approach achieved high accuracy and showed a strong correlation with manual counts results. Lewis et al. (27) proposed a more precise automated workflow, utilizing EfficientNetV2S to classify the uniformly sliced tiles into four categories (optimal, particle, hemodilute and outside), Faster-RCNN to detect cells treated as single category objects within the optimal tiles (i.e., TOIs), and EfficientNetV2L for cell classification. Multiple experiments demonstrated the feasibility of automatically generating DCCs from WSIs. Both methods slice WSIs into tiles, however, these tiles might include areas of cell clumping, overstaining, or blank spaces, resulting in many invalid tiles that need to be filtered out, making the process particularly time-consuming. In practice, the region suitable for DCCs is the body-tail junction area, known as region of interest (ROI).

ROI-based counts methods identify ROIs from WSIs, then extract tiles from these regions for subsequent cell detection and classification. Wang et al. (28) proposed a hierarchical framework that utilizes a multi-resolution pyramid and Cascade R-CNN to identify suitable bounding boxes as ROIs from WSIs. This is followed by another Cascade R-CNN for BMA cell detection and classification within these regions, achieving effective nucleated cell classification and counts analysis from WSIs (28). Su et al. (29) further explored methods for automatically extracting high-quality tile images and accurately locating and identifying nucleated cells, proposing the ROI-BMC-DNNet analysis framework. This framework used a pyramid network and an encoder-decoder to segment ROIs, from which high-quality tiles were then extracted. A tile quality evaluation network and a cell detection network were subsequently used to automatically identify and count nucleated bone marrow cells. Compared to Grid-based counts methods, which slice WSIs into uniformly sized tiles, ROI-based counts methods produce higher quality tiles with less data, resulting in shorter processing times during tile classification.

However, both Tile-based and WSI-based counts methods have significant issues, as they are only applicable to BMA from specific diseases and medical centers. They require extensive labeled data, time, and computational resources, and have poor generalizability. Specifically: (1) Supervised learning issues: 1. Object detection models can only detect cell types known from the dataset, making them suitable only for specific detection tasks. When cell types change, additional labeling of cell bounding boxes is required; 2. Image classification models can only learn limited representation in the current dataset. When applied to different medical centers, they require extensive labeled data and retraining to maintain high accuracy. (2) Efficiency and staining issues: 1. Grid-based counts methods produce a large amount of irrelevant data when slicing WSIs into tiles, which increases the time and computational resources required for tile classification model; 2. BMA from different medical centers may have staining differences, making ROI segmentation and TOI classification based on traditional deep learning models less effective and unsuitable for application in other centers.

In recent years, artificial intelligence has advanced rapidly, particularly in computer vision, where foundation models and self-supervised learning have made significant progress. These models can effectively address issue 1. In terms of foundation models, the Segment Anything Model (SAM), trained on the SA-1B dataset with 11 million images and over 1 billion masks, exhibits strong zero-shot generalization capabilities (30). Even when domain-specific images differ significantly from SAM’s training data, high-precision segmentation results can be achieved by fine-tuning with a small amount of labeled data using prompts. SAM has recently gained considerable attention in the medical imaging field. For instance, methods such as MedSAM (31) and Med-SA (32) have been optimized for general medical images. In the cell segmentation domain, methods like CellSAM (33), Guided Prompting SAM (34), and UNSAM (35) have also shown remarkable effectiveness. In addition, self-supervised learning initially constructed supervisory signals from the interior of images, such as the Jigsaw puzzle prediction task proposed by Doersch et al. (36) and the image rotation prediction task by Noroozi and Favaro (37). Subsequently, contrastive learning became one of the mainstream methods in self-supervised learning, learning useful feature representations by comparing similarities and differences between samples. Notable examples include MoCo proposed by He et al. (38) and SimCLR introduced by Chen et al. (39). Building on these advancements, the MAE model proposed by He et al. uses image masking and reconstruction approach to train feature extractors with strong generalization capabilities from a large amount of unlabeled data, and achieves excellent classification performance by fine-tuning with a small amount of labeled data (40).

For Issue 2, Su et al. (29) addressed the time and computational efficiency issue caused by a large amount of invalid tiles by segmenting ROIs, which occupy only a small portion of WSI, and then extracting high-quality tiles from these regions. However, previous research did not resolve the color differences in BMA smears from different medical centers, nor the significant texture differences among pairs of ROI and non-ROI images, as well as TOI and non-TOI images. Our previously proposed TextureUnet (41) and TCNet (42), which have complex texture feature extraction capabilities, can mitigate the impact of BMA staining differences from a texture perspective. This improves the precision of ROI segmentation and tile classification across different medical centers, effectively enhancing the efficiency of tile classification.

**FIGURE 1:**
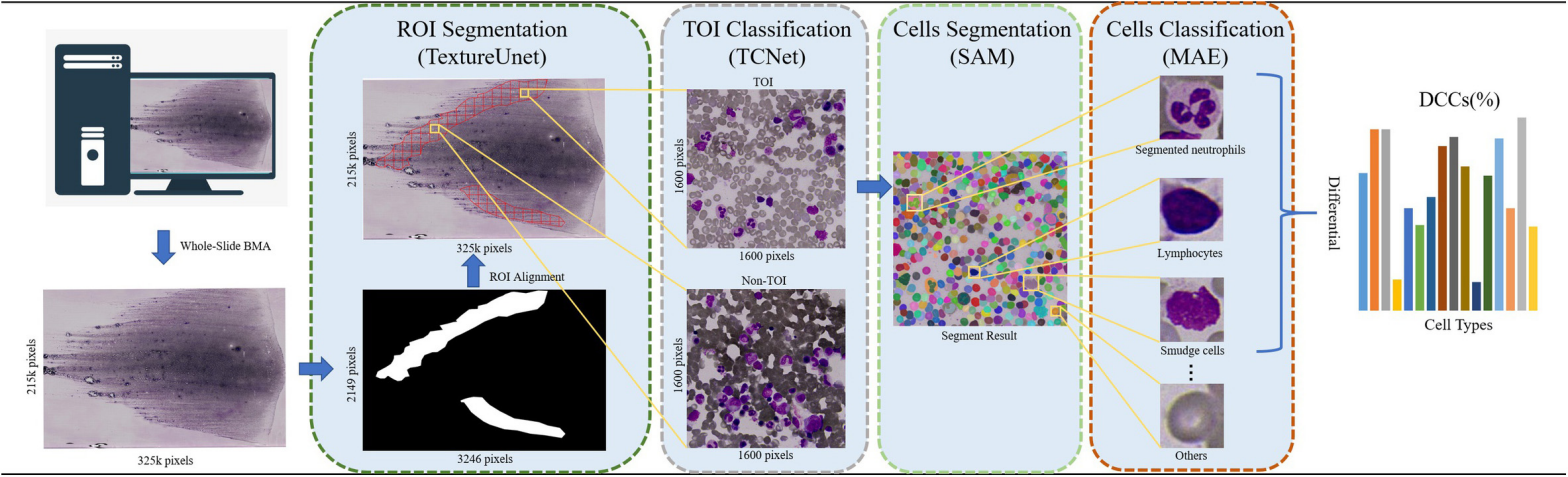
The workflow of the automated framework for DCCs on whole-slide BMA based on the vision foundation model SAM and the self-supervised learning model MAE. The BMA smear is scanned using a 100× oil immersion objective, and its corresponding thumbnail image (with a reduction factor of 100) is processed. The ROI is segmented from the thumbnail using TextureUnet. Tiles are then sliced from the BMA smears corresponding to the ROIs using a grid approach. TCNet classifies the TOIs from these tiles. SAM segments all cells on the TOIs in the everything mode (i.e., SegEvery). MAE is used for cell classification and counts. Finally, a 16-component DCCs histogram is returned.

Based on above, the paper proposed an automated framework for DCCs on whole-slide BMA, leveraging the vision foundation model SAM and the self-supervised learning model MAE (i.e., VFM-SSL-BMADCC-Framework). The framework consists of four stages: ROI segmentation, TOI classification, cell segmentation, and cell classification. (1) ROI Segmentation: In this stage, the framework uses TextureUnet to segment the ROI (i.e., the body-tail junction areas) from the whole-slide BMA thumbnail. (2) TOI Classification: The framework slices the ROI-aligned WSI region into tiles and utilizes TCNet for binary classification to select TOIs. (3) Cell Segmentation: In this stage, SAM, a vision foundation model with strong generalization capabilities, segments all categories of individual cells from the selected TOIs. (4) Cell Classification: The framework uses the high-performance feature extractor trained by the self-supervised learning model MAE to extract features from individual cells and classify and count them according to the required categories. Cell proportions are calculated by dividing the number of each cell type by the total number of cells, resulting in a 16-component DCCs histogram (Figure 1). In summary, our automated framework offers strong generalization, high accuracy, and can provide valuable reference for pathologists across various medical centers.

## 2 Materials and methods

### 2.1 Data source for bone marrow aspirate smears

This work included 40 BMA smears collected from the Hematology Oncology Center Laboratory at Chongqing Cancer Hospital, from 9 February 2022, to 30 April 2024. These smears exhibit varying cell characteristics and pathological features (Supplementary Table 1). The BMA smears were prepared manually by extracting 0.1–0.2 ml of bone marrow fluid from the posterior or anterior iliac crest of patients under local anesthesia using a BMA needle. The fluid was then placed on a glass slide to create 5–6 smears of uniform thickness. After natural drying, 2 smears from each set were selected for Wright-Giemsa staining for routine examination. All BMA smears were scanned using a Bionovation Image Cytometry slide scanning device at 0.1 μm/pixel (100× oil immersion objective), generating WSIs of the BMA smears, with 40 unique images retained. This dataset was named BMA-WSI-Training. Each WSI was annotated by two pathologists with over ten years of experience using Labelme software (43) to mark ROIs and cell masks, followed by tile classification and cell classification using a self-developed annotation program.

### 2.2 Model development and evaluation

#### 2.2.1 ROI segmentation

**FIGURE 2:**
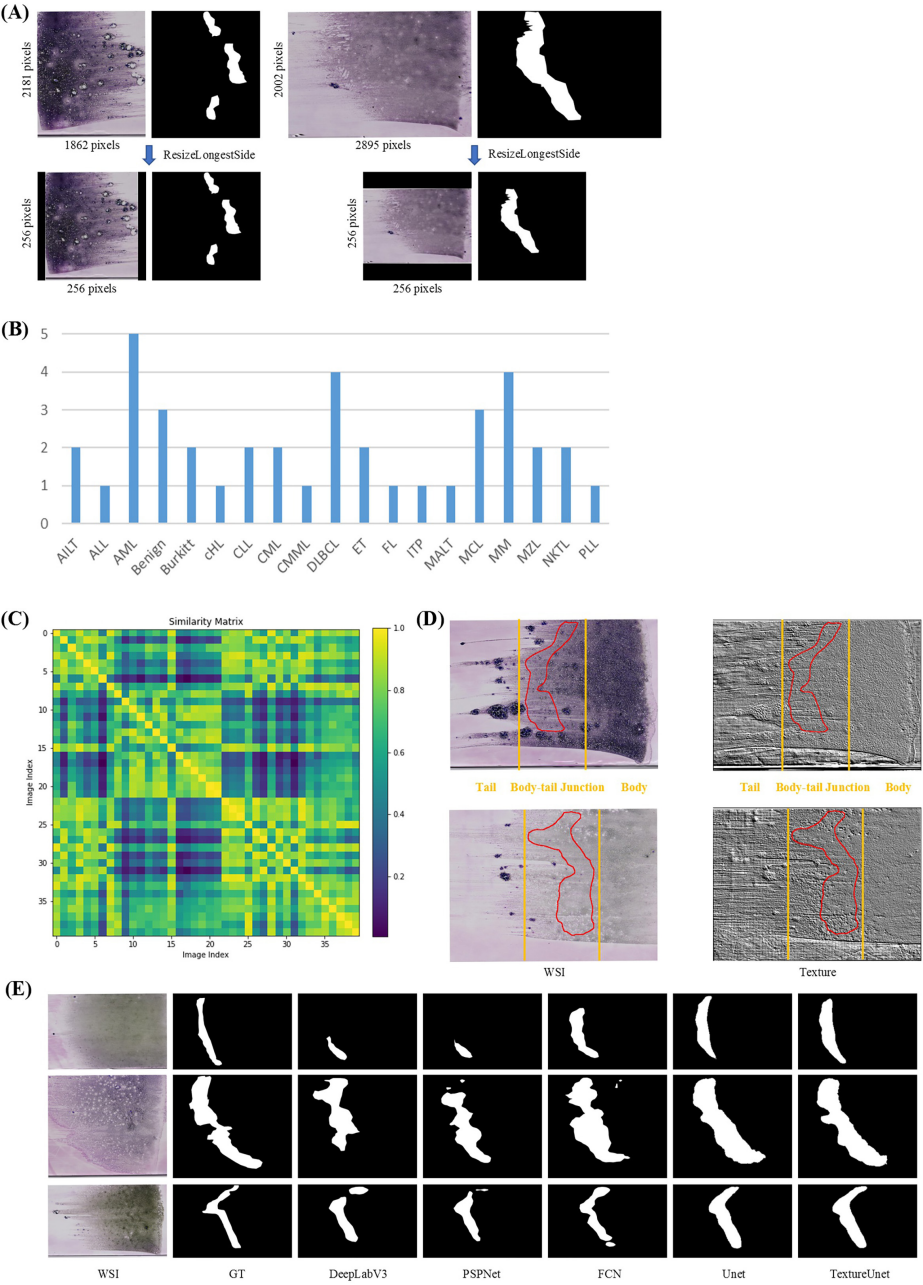
ROI segmentation. **(A)** Zero padding based on ResizeLongestSide: To preserve the original aspect ratio of the WSI thumbnails, the resizing is done by scaling the long side to the target size and applying zero padding on both sides of the short side. **(B)** Disease distribution of ROI-SEG dataset used for training and validation, including 40 WSIs. **(C)** Color similarity matrix of WSIs in the training and validation dataset. The darker the color of the grid, the lower the similarity between the two WSIs corresponding to the grid. Most of the grids in the matrix are dark, indicating significant color differences among WSIs. **(D)** WSI and its corresponding texture feature map. The textures in the body, tail, and body-tail junction areas of the BMA thumbnails exhibit significant differences. **(E)** Actual segmentation results of the model.

In the ROI segmentation stage, WSIs (at least 10k × 10k pixels) from the BMA-WSI-Training dataset were downscaled by a factor of 100 using pyvips in Python, creating a new dataset named the ROI-SEG. This dataset includes different diseases such as AML, Burkitt, CLL, DLBCL, MM (Figure 2B). Pathologists annotated the body-tail junction areas (i.e., ROIs) using Labelme, resulting in 40 annotated ROI masks. All thumbnails and their corresponding ROI masks are resized to 256 × 256 pixels when inputting into the model, using zero-padding based on ResizeLongestSide (Figure 2A).

Whole-slide BMA commonly exhibit staining variability (Figure 2C). Notably, there are distinct texture differences in the body, tail, and body-tail junction areas of the BMA thumbnails (Figure 2D). Given the limited texture feature extraction capability and lower accuracy of traditional segmentation models, we employed our previously proposed TextureUnet (41), which is effective in texture information extraction, to segment ROIs in whole-slide BMA to mitigate the impact of staining variability. The model was trained on the ROI-SEG dataset, which was split into training (80%) and validation (20%). The training parameters included a batch size of 4, Adam optimizer, a learning rate of 1e-4, and a multi-task loss function consisting of cross-entropy loss and dice loss, with a total of 100 epochs. Metrics for evaluation included IoU, Dice and PA. The comparison models used in the ROI segmentation shared the same hyperparameters as TextureUnet.

#### 2.2.2 TOI classification

**FIGURE 3:**
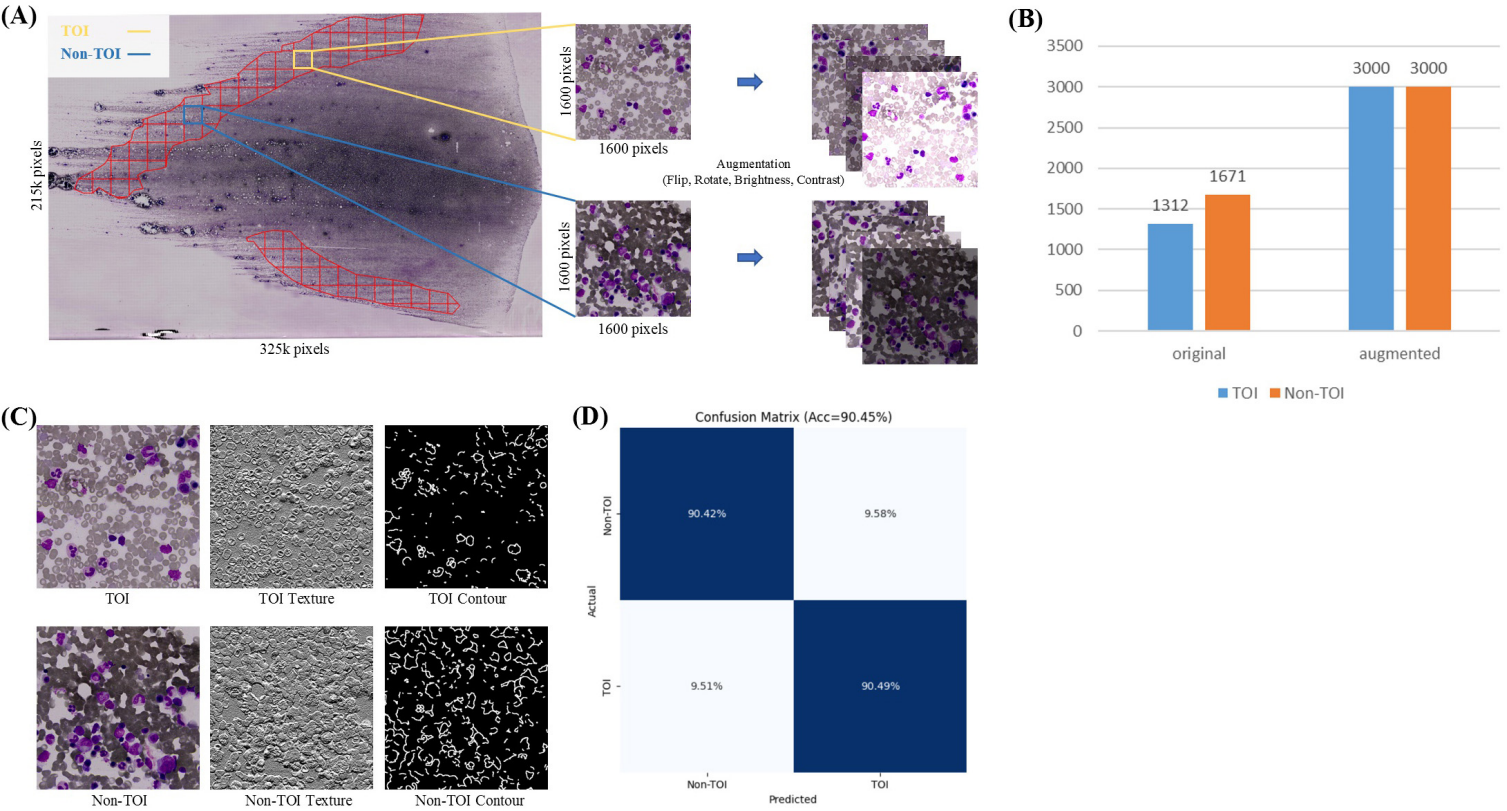
TOI Classification. **(A)** Tiles within the ROI are divided into TOI and non-TOI categories. Data augmentation is performed, including random flipping, rotation, and adjustments to brightness and contrast. **(B)** Number of original and augmented images in the TOI classification stage. **(C)** Texture and contour features of TOI and non-TOI exhibit significant differences. **(D)** Confusion matrix for TOI classification.

In the TOI classification stage, each ROI mask’s corresponding WSI was sliced into non-overlapping tiles based on a grid approach, retaining the tiles within the ROI mask area, while discarding the tiles outside this area. Given the large size of the whole-slide ROI, the retained tiles are numerous enough and include some areas unsuitable for counts (such as blank spaces or excessive staining, Figure 3A). Therefore, further binary classification is needed. Pathologists, following the TOI criteria (bone marrow nucleated cells distributed evenly, thin, without red cell aggregation, over-staining, and cell debris) (26), annotated 1,312 TOIs and 1,671 Non-TOIs from 10 WSIs (Supplementary Table 1) using a custom annotation program. All annotated tiles are 1600 × 1600 pixels in size, containing a sufficient number of cells. The dataset was named TOI-CLS and split into training (80%) and validation (20%). Each tile image and its augmented copies in the training set were resized to 224 × 224 pixels, with horizontal or vertical flips, 90, 180, or 270-degree rotations, and random adjustments to brightness and contrast. This resulted in an augmented training set of 3,000 TOIs and 3,000 Non-TOIs, containing a mix of original and augmented tile images (Figure 3B). The validation set consisted of 598 original tile images.

TOI and Non-TOI images from WSIs have significant differences in texture and contour (Figure 3C). Traditional classification models have weaker generalization abilities and lower accuracy in texture and contour feature extraction. To accurately classify TOIs, we used TCNet (42), which has strong texture and contour feature extraction capabilities and can achieve high accuracy with relatively small datasets. The batch size was set to 128, the Adam optimizer was used with a learning rate of 1e-4, and the loss function was cross-entropy loss. The model was trained for 50 epochs. Metrics for evaluation included Accuracy, Precision, Recall, F1-score and AUROC. The comparison models used in the TOI classification stages shared the same hyperparameters as TCNet.

#### 2.2.3 Cell segmentation

Previous studies have used object detection models to detect tiles of interest within BMA images. However, these methods require extensive labeled data of specific cell types to achieve high accuracy and have limited generalization, making it difficult to apply them to other medical centers. The Segment Anything Model (SAM) (30), developed by Meta AI, addresses this issue by enabling direct segmentation of all cells in TOIs from different medical centers without additional training.

**FIGURE 4:**
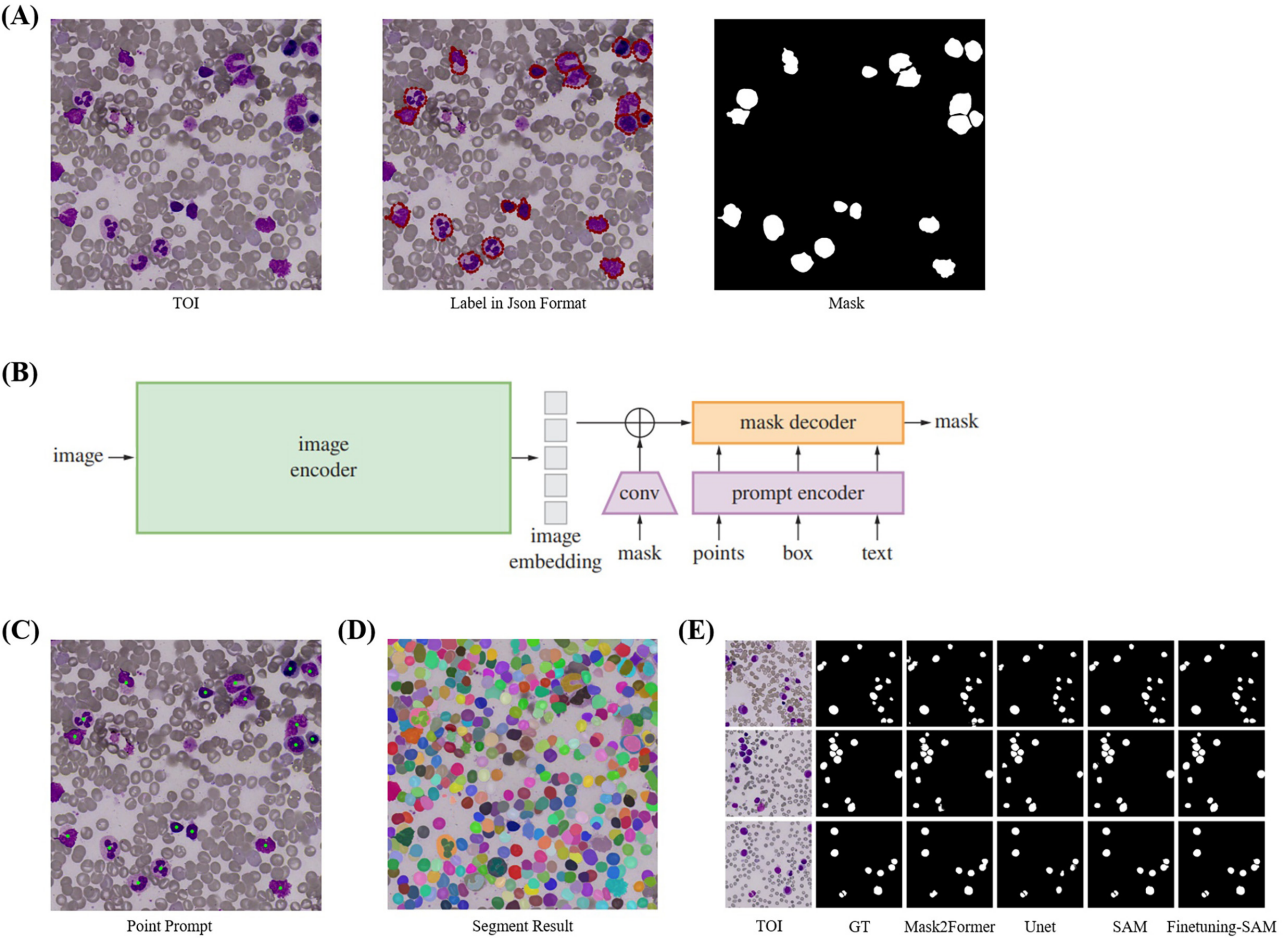
Cell Segmentation. **(A)** TOI and their corresponding annotation information. We annotated the cells using polygons and converted them into masks using Python. **(B)** The model structure of SAM, which includes image encoder, prompt encoder, and mask decoder, and supports four types of prompts: point, box, mask, and text. **(C)** Point prompts, where the geometric center of each cell is used as input for SAM’s point prompts. **(D)** Segmentation results of SegEvery which can segment all foreground objects (i.e., cells). **(E)** Visualization of cell segmentation results from different models.

SAM has three components: an image encoder, a prompt encoder, and a mask decoder, supporting point, box, and text prompts (Figure 4B). Trained on the SA-1B dataset of 11 million images and over 1 billion masks, SAM demonstrates strong segmentation and generalization capabilities. It supports two segmentation methods: SegAny (44), which predicts masks for a single object based on a point or box, and SegEvery (45), which predicts masks for all objects in an image. To segment all cells, we chose ViT-H (632M parameters) (46) as the backbone.

When SAM’s performance in specific domains is suboptimal, fine-tuning can enhance accuracy. Since SAM’s training images are natural images, there are differences compared to bone marrow cell images, so direct segmentation of cells needs improvement. Fine-tuning is necessary with a cell masks dataset. We directly fine-tuned SAM by freezing the official weights of the image encoder and prompt encoder, which handle feature extraction and point prompts effectively, and only updating the decoder weights. We used the geometric center points of cell masks, which precisely locate cells, as foreground points (Figure 4C) for fine-tuning with the SegAny method. In cell segmentation fine-tuning stage, each TOI image annotated with a single cell mask.

During validation and testing, we employed the SegEvery for segmentation (Figure 4D). We did not use box or text prompts because SegEvery is based on point prompts, which improve segmentation performance. In cell segmentation validation stage, each TOI image annotated with all single cell masks, all used for validation.

In a word, our cell segmentation dataset (CELL-SEG) is a subset of the TOI data from the ROI segmentation stage, consisting of 1,000 TOIs. The training dataset includes 800 TOI images, totaling 800 cell masks. The validation dataset includes 200 TOI images with 2,274 cell masks. Pathologists annotated bone marrow nucleated cells in each TOI using polygons in Labelme42. The dataset labels only include foreground 1 and background 0. We then converted the JSON annotation information into cell mask images using Python (Figure 4A). All images and masks were resized to 1024 × 1024 pixels. We set the batch size to 16, used the Adam optimizer with a learning rate of 1e-6, and employed dice loss and BCE loss as the loss functions, training for 30 epochs. The evaluation metrics are Recall50 and Recall75, which measure the proportion of correctly predicted bounding boxes at different IoU thresholds. When applied to other medical centers, due to the relatively fixed morphology of the cells, the fine-tuned SAM demonstrates strong generalization capability and can achieve accurate segmentation without the need for additional fine-tuning. For the comparison models used in the cell segmentation stage, the segmentation models (UNet and Mask2Former) used the same hyperparameters as those applied when fine-tuning SAM. For the detection models (Faster R-CNN and YOLOv3), we set the batch size to 16, used the Adam optimizer with a learning rate of 1e-4, and trained for 30 epochs. Specifically, Faster R-CNN employed BCELoss and Smooth L1 Loss as its loss functions, while YOLOv3 employed BCELoss and MSELoss.

#### 2.2.4 Cell classification

Cell classification is an essential step in BMADCC, and high-precision cell classification models rely on feature extractors. Existing research depends on extensive labeled cell classification data to directly train feature extractors. However, these feature extractors often struggle with varying cell classification tasks and the cell classification labeling process is time-consuming and labor-intensive. In contrast, general-purpose feature extractors can achieve accurate cell classification with only a small amount of labeled data and can be flexibly applied to different cell classification tasks. Therefore, with only a limited amount of labeled cell data, we employ the self-supervised learning model MAE (40) with a ViT-B (46) backbone to accurately classify all cells segmented by SAM in the TOIs, training in two stages: MAE-Cell-Image-Reconstruction stage and Cell-Classification-Fine-tuning stage.

**FIGURE 5:**
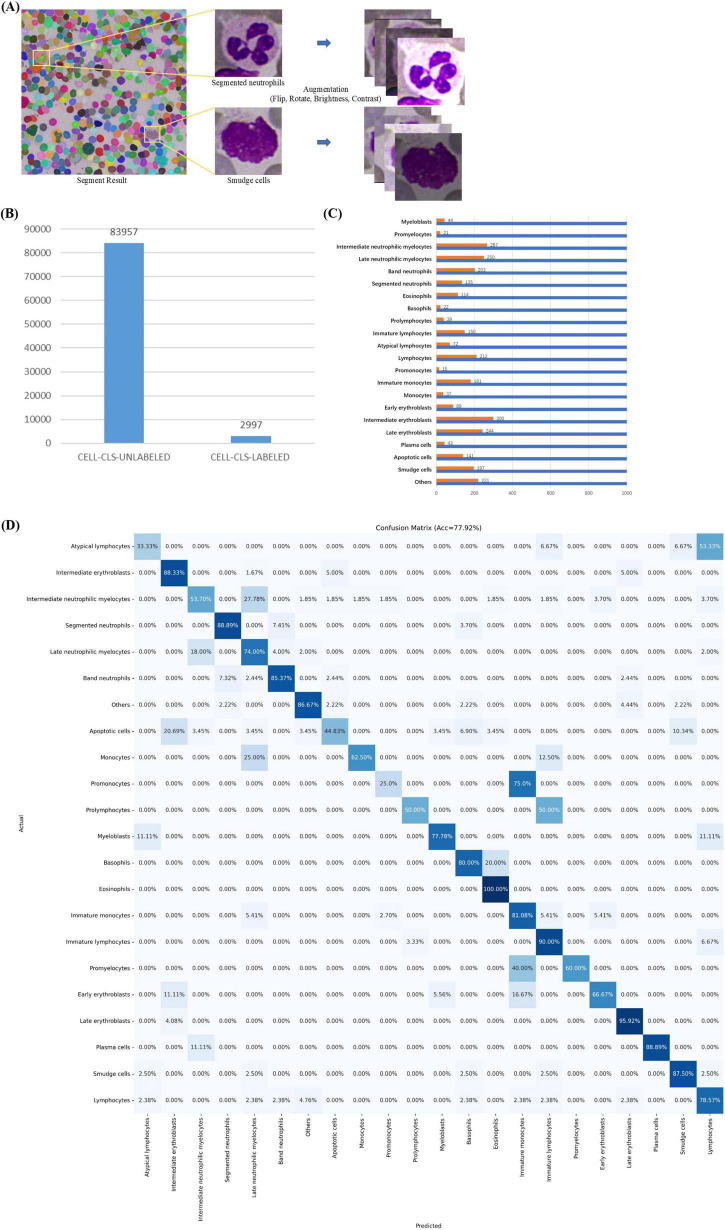
Cell Classification. **(A)** Cell images with box offsets extracted from TOI according to masks, with image augmentation including random flips, rotations, and adjustments to brightness and contrast. **(B)** Data distribution in cell classification stage, including datasets CELL-CLS-UNLABELED and CELL-CLS-LABELED. **(C)** Cell annotation details. **(D)** Confusion matrix for cell classification.

In MAE-Cell-Image-Reconstruction stage, MAE learns general-purpose feature representations through image reconstruction from a large volume of unlabeled cell data. Our unlabeled cell dataset (CELL-CLS-UNLABELED) is based on the cells from the TOIs in the ROI segmentation stage. Using SAM, the cells are segmented and saved as square images of varying sizes with box offsets (Figure 5A).

CELL-CLS-UNLABELED dataset includes 83,957 unlabeled, unaugmented cell images across various diseases such as ALL, AML, AITL, Burkitt, CLL, CML, CMML, cHL, DLBCL, ET, FL, MCL, MALT, MM, NKTL, PLL, and ITP. For image reconstruction, we load the official pre-trained ViT-H weights, set the batch size to 256, use the AdamW optimizer with a learning rate of 1e-3, and employ MSE as the loss function, training for a total of 200 epochs.

In Cell-Classification-Fine-tuning stage, the MAE encoder is used as a general-purpose feature extractor and is fine-tuned with a small amount of labeled cell data to obtain a high-accuracy cell classification model. Our labeled cell dataset (CELL-CLS- LABELED) is similar to the unlabeled cell dataset but does not overlap with it. We selected 22 common cell categories from the union of cell types across the mentioned diseases: apoptotic cell, atypical lymphocyte, band neutrophil, basophil, early erythroblast, eosinophil, immature lymphocyte, immature monocyte, intermediate erythroblast, intermediate neutrophilic myelocyte, late erythroblast, late neutrophilic myelocyte, lymphocyte, monocyte, myeloblast, plasma cell, prolymphocyte, promonocyte, promyelocyte, segmented neutrophil, smudge cell, others (e.g., irrelevant cell, cell fragment, impurity).

CELL-CLS- LABELED dataset includes 2,997 cell images (Figure 5B). Pathologists used a custom labeling program to annotate these cell types. The dataset was split into training (80%) and validation (20%). The training dataset consisted of 2,389 cell images, augmented by horizontally or vertically flipping each cell image, rotating by 90°, 180°, or 270°, and randomly adjusting brightness and contrast (Figure 5A). This process resulted in an augmented training set of 22,000 images (Figure 5C), including both original and augmented cells, while the validation set comprised 608 original cell images. We used the augmented training set for classification fine-tuning. For fine-tuning, we loaded the pre-trained weights based on image reconstruction, set the batch size to 256, used the Adam optimizer with a learning rate of 1e-3, and employed cross-entropy loss as the loss function, training for a total of 200 epochs. We selected Accuracy, Precision, Recall, F1-score and AUROC as evaluation metrics. The comparison models used in the cell classification stages shared the same hyperparameters as classification fine-tuning.

When applied to other medical centers, MAE demonstrates strong classification performance due to its powerful feature extraction capabilities. It allows for effective classification with minimal additional annotation and fine-tuning based on the specific types of cells of interest.

#### 2.2.5 Inter-observer agreement assessment

To ensure the reliability of the annotations used for training and validation, two experienced hematopathologists independently labeled the dataset across four stages: ROI segmentation, TOI classification, cell segmentation, and cell classification.

In the ROI segmentation stage, inter-observer assessment was evaluated using IoU and Dice coefficients, with an average IoU of 0.91 and an average Dice of 0.94; In the TOI classification stage, Cohen’s Kappa coefficient was used to assess inter-observer assessment, yielding a value of 0.88; In the cell segmentation stage, inter-observer assessment was again evaluated using IoU and Dice coefficients, with an average IoU of 0.85 and an average Dice of 0.91; In the cell classification stage, Cohen’s Kappa coefficient was again used, resulting in a value of 0.82; These consistently high values across all four stages demonstrate a strong assessment between the two hematopathologists in their annotations.

### 2.3 Automated framework testing

We used 13 BMA WSIs (BMA-WSI-TESTING) from Chongqing Cancer Hospital that were not used in the training of the automation framework model (Supplementary Table 2) for testing. The staining protocol for all images was Wright-Giemsa staining. The image sizes were similar to those used in the training stage, including diseases such as AML, CLL, MM, and DLBCL. For all test whole-slide BMA, pathologists conducted a 16 categories DCCs according to the guidelines of the International Council for Standardization in Hematology (ICSH) (47), manually counts a total of 300 cells using glass slides. The counted cell types include band neutrophil, basophil, early erythroblast, eosinophil, immature monocyte, intermediate erythroblast, intermediate neutrophilic myelocyte, late erythroblast, late neutrophilic myelocyte, lymphocyte, monocyte, myeloblast, plasma cell, promonocyte, promyelocyte, segmented neutrophil. The other six cell types (apoptotic cell, atypical lymphocyte, immature lymphocyte, prolymphocyte, smudge cell, and others) were not included in the actual count. During testing, the predicted ROI masks were post-processed to ensure the ROI masks matched the shape of the corresponding original BMA WSI thumbnails for subsequent region matching on WSIs.

The 16-component DCCs returned by our automated framework can be directly compared with the manual counts results. We calculated both the Pearson correlation coefficient and the concordance correlation coefficient and designed multiple experiments for correlation analysis.

In clinical analysis, the processing of each slide is divided into two main stages: image preprocessing and intelligent analysis. (1) Image preprocessing: In this stage, WSI is compressed into thumbnails. This step is handled by the Python package pyvips, and its runtime depends on the CPU’s processing speed. All operations were conducted on a server running Ubuntu 23.04, equipped with an AMD EPYC 7542 32-Core processor and 256 GB RAM. On this server, the average time required to tile a single WSI is approximately 13 min. (2) Intelligent analysis: The stages of ROI segmentation, TOI classification, cell segmentation, and cell classification were all performed on an RTX A6000 GPU. The average runtime for each stage was as follows: ROI segmentation—0.28 s; TOI classification—75 s; cell segmentation—302 s; and cell classification—213 s. The significantly shorter runtime for ROI segmentation is due to the fact that it processes only a single image, whereas TOI classification, cell segmentation, and cell classification involve processing hundreds to thousands of images.

During the use of this framework, clinicians are not required to evaluate the quality of intermediate outputs.

### 2.4 Different medical center testing

To evaluate the generalization capability of our automated framework, we conducted experiments using 5 whole-slide BMA images (BMA-WSI-SWH) from Southwest Hospital (Supplementary Table 2). The staining protocol used was the same as that at Chongqing Cancer Hospital, specifically Wright-Giemsa staining, and all cases were diagnosed with DLBCL. Pathologists performed cell classification and counts according to the same 16 categories using the guidelines of the ICSH, consistent with the protocol at Chongqing Cancer Hospital.

We directly utilized the automated framework trained on the dataset BMA-WSI-TRAINING from Chongqing Cancer Hospital to perform inference on the datasets from Southwest Hospital. The output 16-component DCCs were then analyzed for correlation with the manual counts results.

## 3 Results

### 3.1 The ROI segmentation model accurately obtains the regions of interest from bone marrow aspirate thumbnails

In this work, we employed TextureUnet (41), a model with strong texture feature extraction capabilities, to segment ROIs (i.e., the body-tail junction areas) from the BMA thumbnails.

The ROI segmentation model achieved an IoU of 46.19%, Dice score of 63.19%, and PA of 95.38% on the validation set. To validate the effectiveness of TextureUnet, we compared its performance with other classical segmentation networks, including PSPNet (48), FCN (49), DeepLabV3 (50), and U-Net (51), as shown in Table 1. TextureUnet outperformed these models in terms of IoU, Dice, and PA. Due to the higher proportion of non-ROI regions in the annotated masks and the relatively blurry boundaries of the ROI, the PA was high while IoU and Dice scores were lower. The segmentation results of TextureUnet and other models were illustrated in Figure 2E, showing that TextureUnet’s output was quite similar to the manually annotated masks. Overall, the ROI segmentation model effectively identifies ROIs and is suitable for most leukemia and lymphoma cases.

**TABLE 1:** Comparative experiment for ROI segmentation.

Methods	IoU↑	Dice↑	PA↑
DeepLabV3	23.19	35.16	92.20
PSPNet	28.67	42.21	93.50
FCN	30.82	44.56	92.39
U-Net	44.51	61.50	95.23
TextureUnet	**46.19**	**63.19**	**95.38**

The bold values indicate the best performance achieved among the compared methods for each evaluation metric.

The framework proposed in this study was specifically designed with consideration for the potential impact of ROI segmentation on subsequent analysis stages. Therefore, in the TOI classification stage, sliced regions resulting from ROI segmentation are further filtered—only high-quality tiles are forwarded to the cell segmentation stage, while low-quality ones are discarded. In clinical practice, the availability of high-quality smears is generally sufficient. Even if some high-quality regions are missed during segmentation, the remaining tiles are still adequate for downstream analysis. This also aligns with the manual workflow, where pathologists typically select only a small number of high-quality smears for differential cell counts.

### 3.2 TOI classification model accurately selects tiles suitable for cell counts

To accurately identify TOI, we employed TCNet (42), which features texture and contour depth supervision modules. This model demonstrates strong texture and contour feature extraction capabilities on cell tiles and achieves high accuracy with minimal labeled data.

The TOI classification model exhibits excellent performance, as shown in Figure 3D and Table 2. The accuracy was 90.45%, TOI AUROC was 96.81%, Precision was 88.15%, Recall was 90.49%, and F1-score was 89.30%. To validate the effectiveness of TCNet, we compares it with classic classification networks based on CNN, including VGG16 (52), ResNet50 (53), and DenseNet121 (54). TCNet outperformed these models in terms of Accuracy, Precision, Recall, F1-score and AUROC. These results indicate that the TOI classification model can accurately identify regions suitable for BMADCC, regardless of the WSIs’ pathological diagnosis and cell characteristics.

**TABLE 2:** Comparative experiment for TOI classification.

Methods	Accuracy↑	Precision↑	Recall↑	F1-score↑	AUROC↑
VGG16	87.54	86.37	87.90	87.13	95.25
ResNet50	88.13	86.92	88.51	87.71	95.46
DenseNet121	88.92	87.62	89.03	88.32	96.14
TCNet	**90.45**	**88.15**	**90.49**	**89.30**	**96.81**

The bold values indicate the best performance achieved among the compared methods for each evaluation metric.

### 3.3 Cell segmentation model accurately segments all cells

The computer vision foundation model SAM (30) offers strong zero-shot generalization capabilities and achieves higher accuracy without requiring predictions about the class of segmented objects. By using SegEvery (45), we can segment all cells within the TOIs. When segmentation results are suboptimal, SAM can be fine-tuned to improve accuracy. In this framework, SAM’s output is filtered using Non-Maximum Suppression (NMS) (55) to remove duplicate masks.

SAM demonstrated impressive segmentation performance. We compared SAM with several object detection, semantic segmentation, and instance segmentation models. Since SAM and other segmentation models return masks rather than bounding boxes, we used the bounding boxes derived from the masks to calculate IoU. The evaluation metrics are Recall50 and Recall75, which measure the proportion of correctly predicted bounding boxes at different IoU thresholds. As shown in Table 3, the fine-tuned SAM outperformed current cell detection [Faster-RCNN (56), YOLOv3 (57)], semantic segmentation [U-Net (51)] and instance segmentation models [Mask2Former (58)] in terms of Recall50 and Recall75. The segmentation accuracy of fine-tuned SAM also exceeded that of the original SAM. Overall, SAM is capable of accurately segmenting all cells in TOI from various diseases. As shown in Figure 4E, the fine-tuned SAM demonstrates a significant advantage over the baseline SAM and other segmentation models. While the original SAM already exhibits strong zero-shot segmentation capabilities, it occasionally fails to delineate precise cell boundaries, particularly in regions with blurred edges. In contrast, the fine-tuned SAM can segment cells of various morphologies with greater accuracy, outperforming the other comparative models.

**TABLE 3:** Comparative experiment for cell segmentation.

Methods	Recall50↑	Recall75↑
Faster-RCNN	96.52	93.35
YOLOv3	97.60	92.39
Unet	96.14	93.18
Mask2Former	86.86	68.55
SAM	99.77	98.37
Fintuning-SAM	**99.77**	**99.01**

The bold values indicate the best performance achieved among the compared methods for each evaluation metric.

Although SAM demonstrates strong zero-shot performance in cell segmentation tasks, our results indicate that fine-tuning SAM on domain-specific datasets can still bring non-negligible benefits. In high-precision segmentation (IoU ≥ 0.75), the 0.64% improvement suggests that fine-tuning enhances the model’s ability to capture finer object boundaries and structural details. This is particularly critical in medical image analysis, where accurate localization directly affects the reliability of subsequent diagnoses.

Moreover, considering that segmentation errors under high IoU thresholds may propagate to downstream tasks (such as cell counting or subtype classification), the improved segmentation accuracy through fine-tuning justifies the additional training effort in high-reliability clinical scenarios.

From a hematopathologist’s perspective, the subtle improvement in boundary recognition achieved by the fine-tuned model can reduce both over-segmentation and under-segmentation, minimizing the need for manual correction and increasing overall diagnostic efficiency and confidence. This is especially valuable in regions with complex bone marrow cell morphology and densely packed adjacent cells, where precise boundary detection helps distinguish overlapping cells more effectively—allowing pathologists to complete reviews and confirmations more rapidly and accurately.

### 3.4 Cell classification model achieves accurate classification with limited annotation data

In complex scenarios like cell image analysis, using a classification model alone after cell detection can significantly improve classification accuracy. Considering the high time cost of cell classification annotation, we use the self-supervised learning-based MAE (40), which achieves good classification results with only a small amount of labeled data.

We compared MAE with convolutional neural networks Resnext101_32 × 8d (59) and ViT-B (46), noting that their parameter sizes and accuracy on ImageNet (60) are similar. As shown in Table 4, compared to Resnext101_32 × 8d and ViT-B, the self-supervised MAE achieves excellent classification performance with minimal labeled data for fine-tuning. ViT-B performs worse than Resnext101_32 × 8d because convolutional neural networks generally perform better on smaller datasets. Moreover, since MAE’s reconstruction stage is self-supervised and does not require labeled data, it directly reconstructs on a large unlabeled dataset CELL-CLS-UNLABELED before fine-tuning on a small labeled dataset CELL-CLS- LABELED, making it more generalizable in practical scenarios.

**TABLE 4:** Comparative experiment for cell classification.

Methods	Natural image pre-trained weights	CELL-CLS-UNLABELED	CELL-CLS-LABELED	Accuracy↑
ViT-B	√	×	√	66.75
Resnext101_32 × 8d	√	×	√	77.16
MAE	√	√	√	**77.92**

The bold value indicates the best performance achieved among the compared methods for each evaluation metric.

The MAE-based cell classification model showed robust performance across 22 cell types, with an average AUROC value exceeding 0.95 (Figure 5D and Table 5). Most cell categories achieved accuracy, precision, recall, F1-score, and AUROC scores above 0.8, with AUROC values over 0.9, and an average accuracy of 77.92%, reflecting its strong cell classification capabilities.

**TABLE 5:** Classification results of the cell classification model.

Cell type	AUROC↑	Precision↑	Recall↑	F1-score↑
Early erythroblasts	95.93	75.00	66.67	70.59
Intermediate erythroblasts	97.78	84.13	88.33	86.18
Late erythroblasts	98.34	87.04	95.92	91.26
Monocytes	99.50	83.33	62.50	71.43
Promonocytes	85.93	33.52	25.00	28.64
Immature monocytes	98.90	76.92	81.08	78.95
Lymphocytes	97.44	68.75	78.57	73.33
Prolymphocytes	88.23	80.00	50.00	61.54
Immature lymphocytes	98.01	71.05	90.00	79.41
Atypical lymphocytes	98.27	62.50	33.33	43.48
Basophils	87.14	40.00	80.00	53.33
Eosinophils	99.91	88.46	100.00	93.88
Smudge cells	96.94	87.50	87.50	87.50
Myeloblasts	96.10	77.78	77.78	77.78
Promyelocytes	98.70	100.00	60.00	75.00
Intermediate neutrophilic myelocytes	91.81	72.50	53.70	61.70
Late neutrophilic myelocytes	95.01	60.66	74.00	66.67
Band neutrophils	96.79	87.50	85.37	86.42
Segmented neutrophils	97.73	85.71	88.89	87.27
Plasma cells	91.21	100.00	88.89	94.12
Apoptotic cells	91.73	68.42	44.83	54.17
Others	90.45	88.15	90.49	89.31

We also identified certain limitations of our framework in detecting specific cell types. Therefore, we have implemented a confidence-based warning mechanism within the model: when the prediction confidence for any cell type is below 60%, a mandatory review by a pathologist is triggered. Additionally, if the model assigns a misclassification rate ≥ 10% to more than one cell type, an “automatic rescreening” alert is generated for manual verification.

Briefly, the cell recognition performance of the proposed framework can be categorized into three levels:

(1) High-confidence recognition (accuracy > 80%):

The framework performs robustly in identifying a variety of mature cell types, including segmented/band neutrophils, eosinophils, basophils, intermediate/late erythroblasts, plasma cells, and smear cells. These results support the diagnostic process for chronic myeloproliferative neoplasms (CMPN), and plasma cell disorders and reliably distinguish nucleated bone marrow cells from staining artifacts.

(2) Moderate-confidence recognition (accuracy 60–70%):

For some cell types such as immature monocytes and atypical lymphocytes, the model serves as a valuable preliminary screening tool. To mitigate diagnostic risks: If the misclassification rate exceeds 10%, the system automatically issues a “manual rescreening” alert. If prediction confidence is below 60% for any category, expert review is mandated.

(3) Low-confidence recognition or high-overlap categories:

Monoblast vs. immature monocyte (Row 10, Column 15, error rate: 75%): These cells show substantial morphological overlap (e.g., size, N/C ratio), differing mainly in chromatin detail. As reported by Osman et al. (61), distinguishing these subtypes morphologically is inherently difficult. Notably, both are clinically regarded as equivalent in WHO classification due to similar prognostic value (62).

Atypical lymphocyte vs. lymphocyte (Row 1, Column 22, error rate: 53.33%): Atypical lymphocytes typically exhibit only subtle morphological changes and are often present in low proportions. Given their close resemblance to normal lymphocytes, even expert-level inter-observer agreement is about 60% (62). Flow cytometry is often required for definitive classification.

Prolymphocyte vs. Immature Lymphocyte (Row 11, Column 16, error rate: 50%): These cells are similar in size and nuclear/cytoplasmic morphology, with only minor differences in cytoplasmic granules and nucleoli. As WHO guidelines (63), they are considered equivalent in the context of diagnosing acute lymphoblastic leukemia.

Promyelocyte vs. immature monocyte (Row 17, Column 15, error rate: 40%): Promyelocytes and immature monocytes are similar in size, making morphological distinction challenging—even for experienced hematopathologists (64, 65). These cells often require cytochemical staining or flow cytometry for accurate classification. We plan to improve accuracy in future iterations by expanding the labeled training dataset.

These considerations provide a clearer understanding of where model limitations may affect clinical interpretation and where errors are less likely to impact diagnosis due to biological or clinical equivalence.

### 3.5 High correlation between automated framework and manual differential cell counts

**FIGURE 6:**
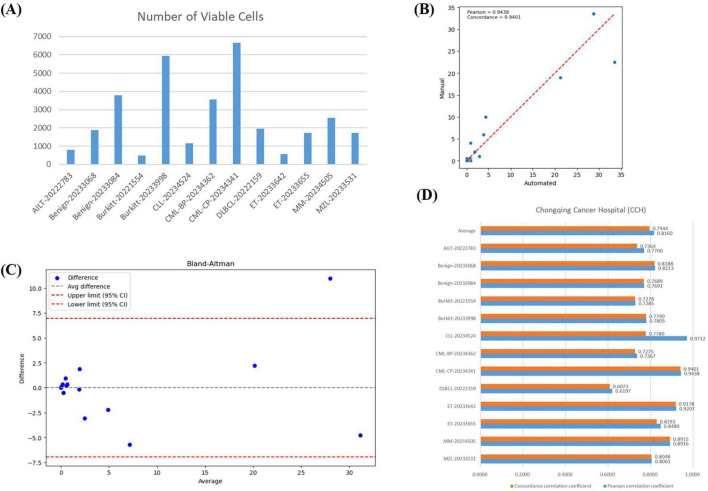
Correlation analysis. **(A)** The number of effective cells segmented from each WSI in the BMA-WSI-TESTING dataset. **(B)** Regression plot of DCCs from manual counts and the automated framework for CML-CP-20234341. The closer the points in the two regions are to the red line, the more related they are. **(C)** Bland-Altman plot of DCCs from manual counts and the automated framework for CML-CP-20234341. Most points within the upper and lower limits represent small differences. **(D)** Pearson correlation coefficient and concordance correlation coefficient for each WSI in BMA-WSI-TESTING dataset.

Our VFM-SSL-BMADCC-Framework demonstrated good performance on the validation set after training. To verify the framework’s generalization capability and the high correlation between the results and manual analysis, we used 13 whole-slide BMA images from Chongqing Cancer Hospital (Supplementary Table 2) for comparative analysis. The number of effective cells segmented from each WSI was shown in Figure 6A, with an average of 3,042 cells. Figure 6B showed the regression plot of the DCCs from manual counts and the automated framework for CML-CP-20234341 (results for other WSIs were provided in Supplementary Figure 1), with a Pearson correlation coefficient of 0.9438 and a concordance correlation coefficient of 0.9401, indicating a very strong correlation between the two. Figure 6C showed the Bland-Altman plot for the DCCs from manual counts and the automated framework for CML-CP-20234341, most differences within the limits of agreement, indicating good consistency between the two (results for other WSIs were provided in Supplementary material). The Pearson and concordance correlation coefficients for all test WSIs were shown in Figure 6D, with an average close to 0.8. Overall, the automated framework proposed in this paper provided DCCs that were highly correlated and consistent with manual DCCs.

### 3.6 The automated framework is applicable to bone marrow cell counts on whole-slide images from different medical centers

Previous studies have proposed frameworks that are only applicable to BMADCC within the same medical center, exhibiting poor generalization and limiting practical application and dissemination. To test the generalization capability of the automated framework across different medical centers, we conducted experiments using 5 WSIs from Southwest Hospital (SWH) (Supplementary Table 2).

**FIGURE 7:**
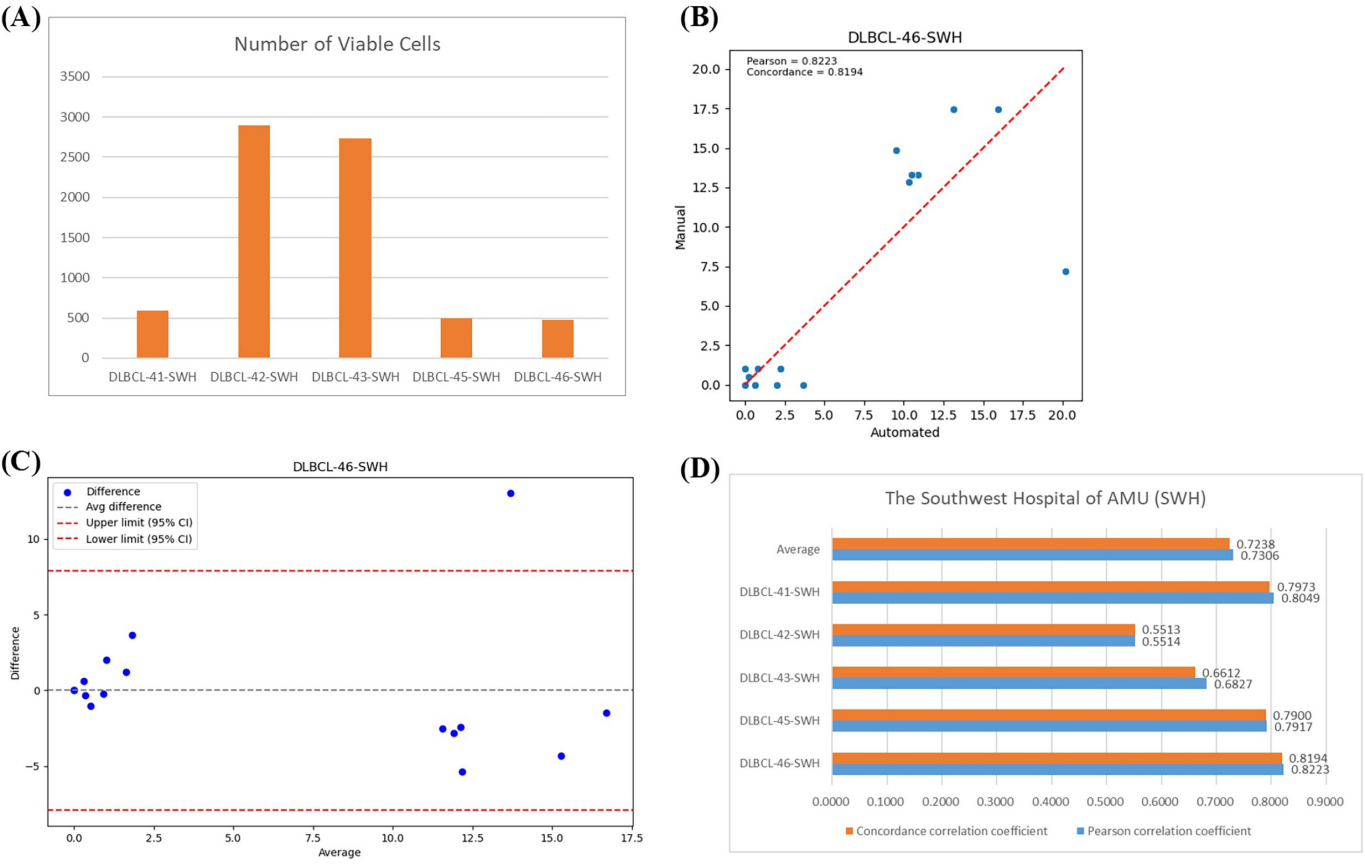
Correlation Analysis Across Different Medical Centers. **(A)** The number of effective cells segmented per WSI from SWH. **(B)** Regression plot of the cell proportion for manual counts versus automated framework counts for DLBC-46-SWH. **(C)** Bland-Altman plot of the cell proportion for manual counts versus automated framework counts for DLBC-46-SWH. **(D)** Pearson correlation coefficient and agreement correlation coefficient for each WSI from SWH.

In the SWH experiment, the average number of effective cells per slide was 1,437 (Figure 7A). Figure 7B showed the regression analysis between manual counts and automated framework counts for the DLBC-46-SWH sample, with a Pearson correlation coefficient of 0.8223 and an agreement correlation coefficient of 0.8149, indicating strong correlation between the two methods. The Bland-Altman plot further confirmed that most differences fall within the agreement limits (Figure 7C), indicating good consistency in the counts results. The average Pearson correlation coefficient and agreement correlation coefficient for all SWH test samples were approximately 0.73 (Figure 7D), suggesting consistent performance of the automated framework across different samples. Overall, the proposed automated framework effectively performed BMADCC across different medical centers, showing high correlation and consistency with manual results.

## 4 Discussion

Most previous studies on bone marrow cell classification and counts relied on traditional machine learning or deep learning algorithms, requiring training on specific large annotated datasets to achieve high accuracy. When the cell detection task changes or when applying to new datasets at other centers, traditional models often see a significant drop in accuracy due to staining differences in BMA and variations in cell types. This necessitates re-annotating data and retraining the models to maintain high accuracy, making it difficult to generalize. With the continuous advancement of AI technology, there have been significant developments in the application of foundation models and self-supervised learning in computer vision. The self-supervised learning model MAE was trained on a large number of unannotated target domain images through masking and reconstruction, resulting in a backbone that serves as a feature extractor with strong generalization capabilities. With only a small amount of annotated data from the target domain for fine-tuning, it can achieve good classification results. The visual foundation model SAM was trained on the SA-1B dataset with 11 million images and over 1 billion masks, exhibiting powerful zero-shot generalization capabilities. Even with a small amount of domain-specific images that differ significantly from the SAM training data, high-precision segmentation results can be achieved by fine-tuning with a few annotated data via prompts. Since there may be staining differences when preparing BMA smears at different medical centers, classic image segmentation and classification models may not be directly applicable to other centers. We use TextureUnet and TCNet, which have complex texture feature extraction capabilities, to reduce the impact of BMA staining differences and accurately segment and classify BMA thumbnails and tiles from different medical centers. Based on this, we propose an automated framework for BMA cell classification and counts using the visual foundation model SAM and the self-supervised learning model MAE (i.e., VFM-SSL-BMADCC-Framework). In the cell detection or segmentation stage, compared to traditional target detection algorithms like Faster-RCNN, we can segment all cells in the TOIs using the original SAM pre-trained model, which is more accurate than traditional target detection algorithms. We can also further fine-tune SAM to achieve better segmentation accuracy. On the other hand, previous methods often involved directly slicing the original image into tiles to find the TOI on WSIs, resulting in many non-TOI tiles. By using TextureUnet to perform region segmentation on the original image to obtain ROIs, we achieve higher quality tiles within that region and reduce the amount of data. This results in shorter processing time for TOI classification. Cell classification annotation is time-consuming and highly dependent on the technician’s experience level. We use the self-supervised learning model MAE for cell classification, which achieves good results with only a small amount of annotated data.

Our proposed framework is applicable to various types of leukemia and lymphoma. The process begins with the ROI segmentation model TextureUnet, which segments the ROI from the thumbnail of the whole-slide BMA image that requires cell counts. Then, tiles are obtained from the corresponding ROI of the whole-slide BMA image. These tiles are filtered for TOI using the TOI classification model TCNet. Next, all cells within the TOI are segmented using the cell segmentation model SAM. Finally, the cells are classified using the cell classification model MAE, and a histogram of cell proportions is generated. All four stages of our models demonstrate strong performance, and DCCs on BMA-WSI-TESTING dataset, which was not used for model training, are highly consistent with the manual results.

The VFM-SSL-BMADCC framework demonstrates high accuracy, strong generalization, and low annotation dependency, making it highly adaptable to real-world clinical workflows. Specifically, the model can be integrated in the following ways: (1) As a pre-screening tool: It can efficiently process whole-slide images, prioritize abnormal cells for review, and significantly reduce manual workload, thereby improving clinical throughput. (2) As a second reader: It supports collaborative diagnostics, enhancing accuracy and consistency across the diagnostic workflow. For rare or easily missed cell types, the deep learning model enables precise localization and identification, improving detection rates and reducing missed diagnoses. (3) As a training aid: The system provides standardized references, expands case libraries, and offers real-time feedback to accelerate the learning curve for junior pathologists. We believe these integrated pathways will facilitate the practical adoption of the model in routine hematopathology practice.

Our automated framework still has aspects for optimization in future work: (1) Cell Segmentation Stage: SegEvery may also segment unwanted cells or objects, such as red blood cells. We can design a SAM with automatic point prompts to accurately segment each nucleated cell in the bone marrow. (2) Other Stages: As the performance of deep learning models continues to improve, we can apply more advanced models in the other three stages to achieve better results.

In summary, VFM-SSL-BMADCC-Framework significantly reduces the time required for cell classification and counts while ensuring accuracy and consistency. It demonstrates strong generalization capabilities.

## Data Availability

The original contributions presented in this study are included in this article/Supplementary material, further inquiries can be directed to the corresponding authors.
