# Supplementary material for: VFM-SSL-BMADCC-Framework: vision foundation model and self-supervised learning based automated framework for differential cell counts on whole-slide bone marrow aspirate smears
Source: Front Med (Lausanne). 2025 Sep 24;12:1624683. doi: 10.3389/fmed.2025.1624683 (PMC12504877; doi:10.3389/fmed.2025.1624683)
Supplement: Supplementary file 1 [file Data_Sheet_1.docx]

Supplementary Material

# Supplementary Figures and Tables

## Supplementary Figures


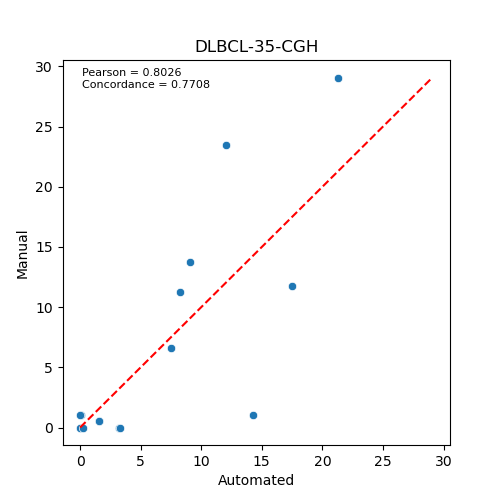

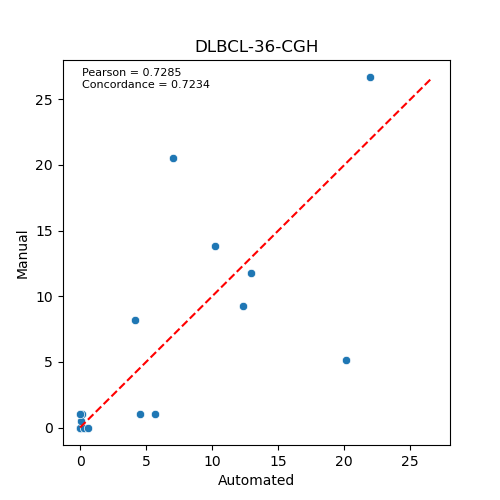

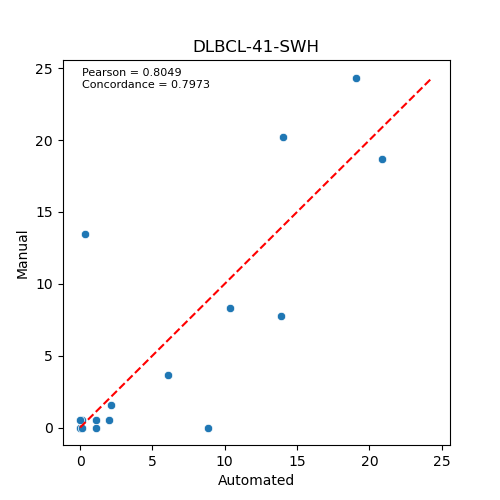

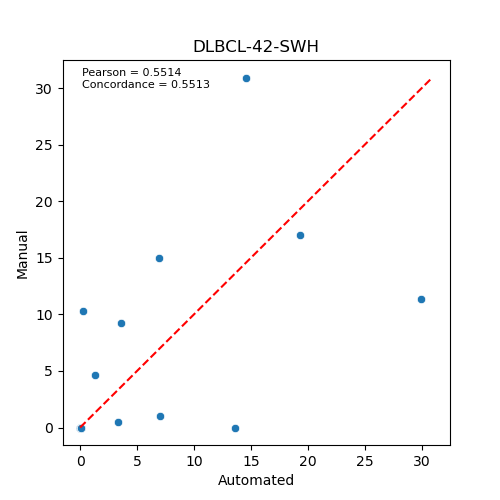

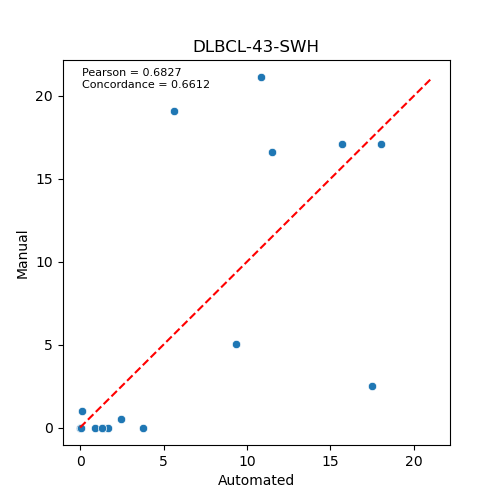

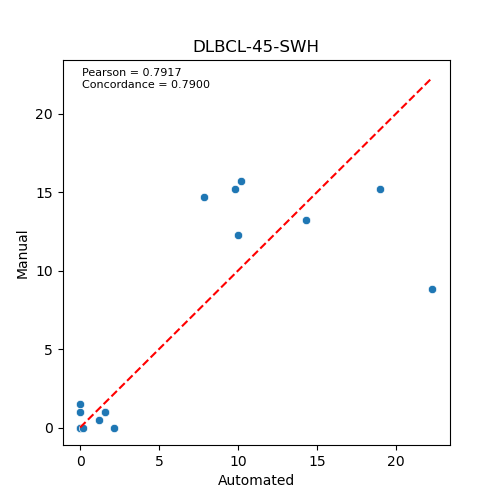

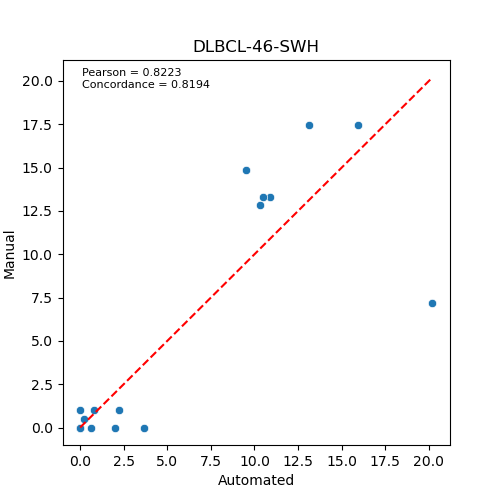

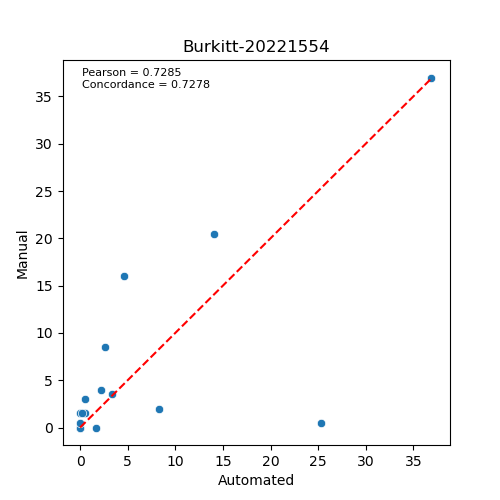

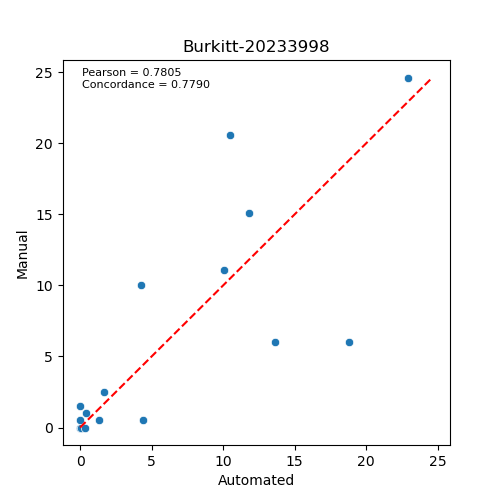

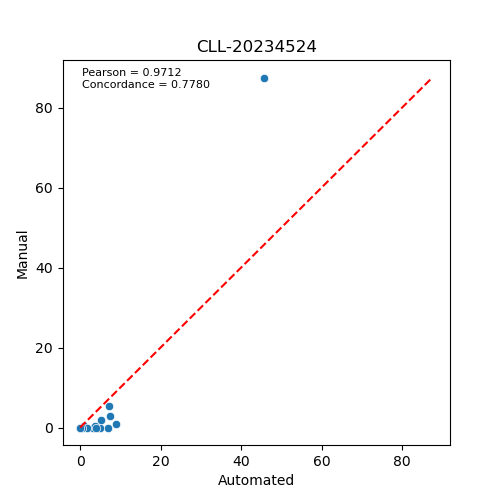

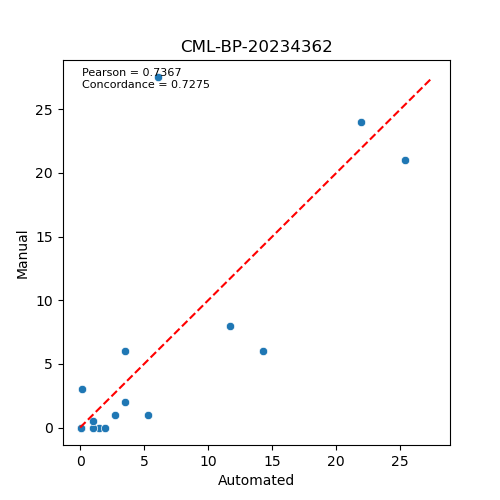

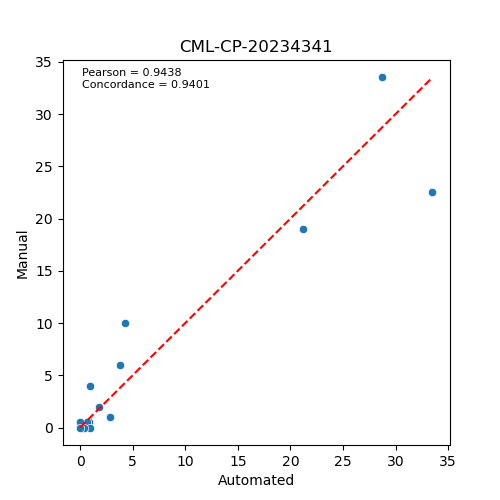

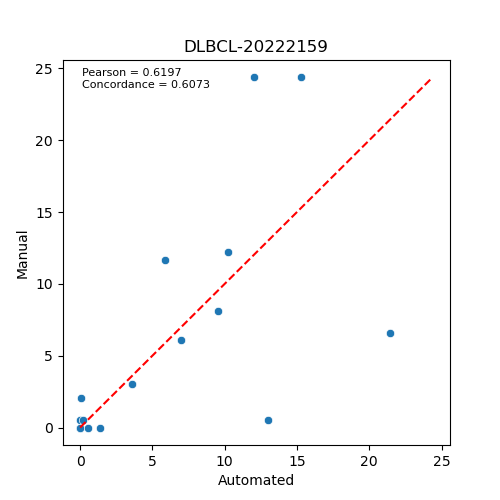

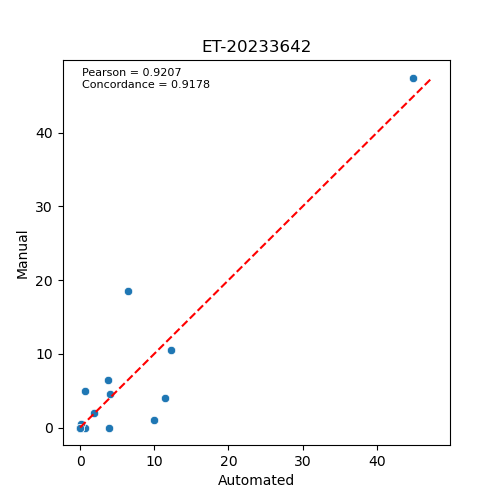

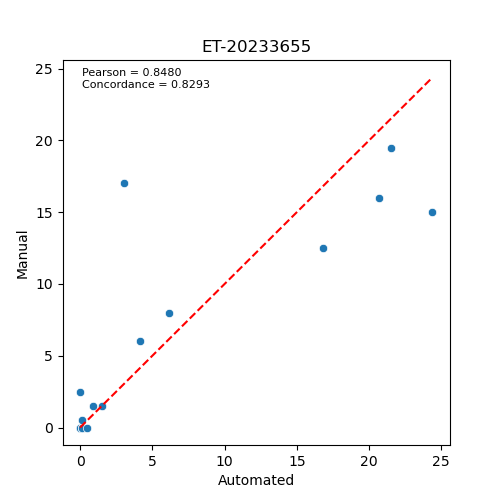

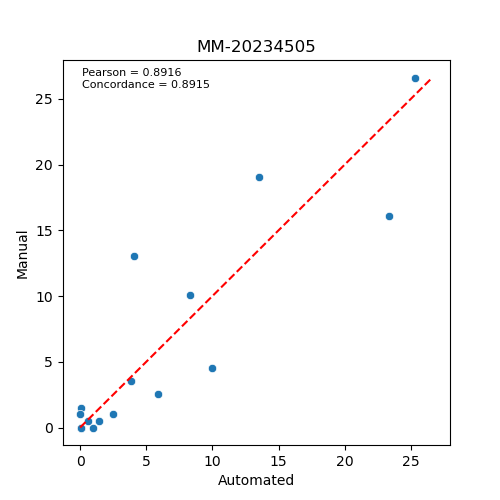

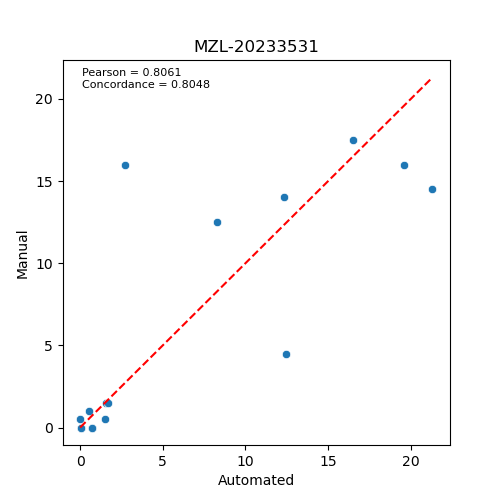

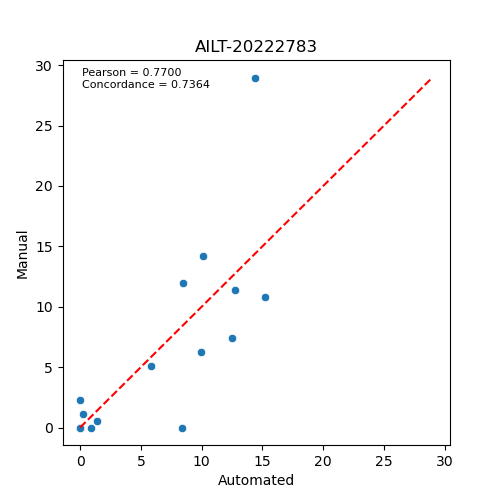

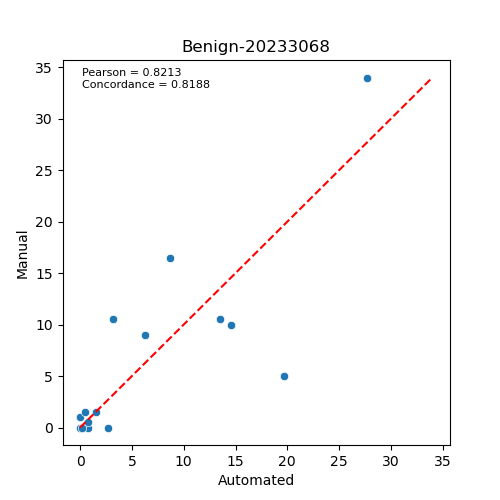

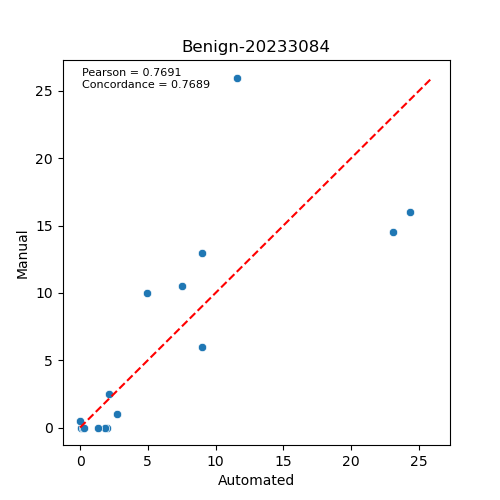


**Supplementary Figure 1.** Regression plot of DCCs from manual counts and the automated framework.

## Supplementary Tables

**Supplementary Table 1.** Information of BMA-WSI-Training dataset.

| Name of WSI | Diseases | ROI Segmentation | TOI Classification | Cell Segmentation | Cell Classification (unlabelled) | Cell Classification (labelled) |
| --- | --- | --- | --- | --- | --- | --- |
| 20233500-1-14 | DLBCL | √ |  |  | √ |  |
| 20233560-22 | CLL | √ | √ |  |  | √ |
| 20233572-23 | DLBCL | √ |  |  | √ |  |
| 20233575-1-20 | Benign | √ |  |  | √ |  |
| qp-20233974-18 | MALT | √ |  |  |  | √ |
| qp-20233975-17 | cHL | √ |  |  | √ |  |
| qp-20233998-15 | Burkitt | √ |  |  |  | √ |
| qp-20234019-2-32 | MCL | √ |  |  | √ |  |
| qp-20234111-59 | FL | √ |  |  | √ |  |
| 20233445-2-11 | MM | √ | √ | √ |  | √ |
| 20233624-1-26 | DLBCL | √ | √ | √ |  | √ |
| qp-20234000-21 | AML | √ | √ |  |  | √ |
| qp-20234019-1-31 | MCL | √ | √ | √ |  |  |
| qp-20234027-28 | NK/T-cell lymphoma | √ | √ | √ |  |  |
| qp-20234047-41 | MM | √ | √ | √ |  |  |
| qp-20234062--46 | AML | √ | √ | √ |  |  |
| qp20234101-55 | MM | √ | √ |  |  |  |
| qp-20234056-39 | MCL | √ |  |  |  |  |
| qp-20234049-2-40 | Extranodal NK/T-cell lymphoma | √ |  |  |  |  |
| qp-20234045-42 | ITP | √ |  |  |  |  |
| qp-20234042-47 | PLL | √ |  |  |  |  |
| qp-20234021-23 | DLBCL | √ |  |  |  |  |
| 20233460-1-10 | MM | √ |  |  |  |  |
| CML20245057M1 | CML | √ |  |  |  |  |
| CML-20245181 | CML | √ |  |  | √ |  |
| CMML-20245383 | CMML | √ |  |  | √ |  |
| Z-20234152 | Benign | √ |  |  | √ |  |
| Z-20234184 | Benign | √ | √ |  | √ |  |
| ET-20245199 | ET | √ |  |  | √ |  |
| ET-20245142 | ET | √ |  |  | √ |  |
| AILT-20234216 | AILT | √ |  |  | √ |  |
| AILT-20244772 | AILT | √ |  |  | √ |  |
| 20221657-CLL | CLL | √ |  |  | √ |  |
| Burkitt-20222237 | Burkitt | √ |  |  | √ |  |
| MZL-20244861 | MZL | √ |  |  | √ |  |
| MZL-20222900 | MZL | √ |  |  | √ |  |
| AML-M2-20245451 | AML | √ |  |  | √ |  |
| AML-M3-20244906 | AML | √ |  |  | √ |  |
| 20245563-all | ALL | √ |  |  |  | √ |
| M5-20245451 | AML | √ |  |  |  | √ |

**Supplementary Table 2.** Information of BMA-WSI-Testing and BMA-WSI-SWH datasets.

| Name of WSI | Diseases | Hospital |
| --- | --- | --- |
| Burkitt-20221554 | Burkitt | Chongqing Cancer Hospital (CCH) |
| Burkitt-20233998 | Burkitt | Chongqing Cancer Hospital (CCH) |
| CLL-20234524 | CLL | Chongqing Cancer Hospital (CCH) |
| CML-BP-20234362 | CML | Chongqing Cancer Hospital (CCH) |
| CML-CP-20234341 | CML | Chongqing Cancer Hospital (CCH) |
| DLBCL-20222159 | DLBCL | Chongqing Cancer Hospital (CCH) |
| ET-20233642 | ET | Chongqing Cancer Hospital (CCH) |
| ET-20233655 | ET | Chongqing Cancer Hospital (CCH) |
| MM-20234505 | MM | Chongqing Cancer Hospital (CCH) |
| MZL-20233531 | MZL | Chongqing Cancer Hospital (CCH) |
| Benign-20233068 | Benign | Chongqing Cancer Hospital (CCH) |
| Benign-20233084 | Benign | Chongqing Cancer Hospital (CCH) |
| AILT-20222783 | AILT | Chongqing Cancer Hospital (CCH) |
| DLBCL-41-SWH | DLBCL | The Southwest Hospital of AMU (SWH) |
| DLBCL-42-SWH | DLBCL | The Southwest Hospital of AMU (SWH) |
| DLBCL-43-SWH | DLBCL | The Southwest Hospital of AMU (SWH) |
| DLBCL-45-SWH | DLBCL | The Southwest Hospital of AMU (SWH) |
| DLBCL-46-SWH | DLBCL | The Southwest Hospital of AMU (SWH) |
